# Intermittent Food Absence Motivates Reallocation of Locomotion and Feeding in Spotted Munia (*Lonchura punctulata*)

**DOI:** 10.5334/jcr.af

**Published:** 2015-06-08

**Authors:** Amrita Srivastava, Shalie Malik, Garima Yadav, Sangeeta Rani

**Affiliations:** DST IRHPA Center for Excellence in Biological Rhythm Research, Department of Zoology, University of Lucknow, India

**Keywords:** Feeding, Food deprivation, Locomotion, Reallocation, Spotted Munia

## Abstract

**Background:** Daily feeding and locomotion are interrelated behaviours. The time spent in feeding and rate of food intake depends on food availability. In low food condition, the birds would show intense movement (locomotion) for a longer time throughout the day however during abundant food supply they may chose higher activity and food intake in the morning and evening only. In the present study we hypothesized that in Spotted Munia (*Lonchura punctulata*), intermittent food availability during day would reallocate their interrelated behaviors, the feeding (food intake) and locomotor activity patterns.

**Methods:** Two groups of birds (*N* = 6 each) were kept individually in activity cages under 12L:12D. Group 1 (Control; C) had *ad libitum* food but group 2 (Treatment; T) had food for 6 hours only (2 h presence followed by 2 h absence; 2P:2A) during 12 hour light period. In the first week, group 2 received food with ‘lights on’ (TI; ZT 0–2, 4–6 and 8–10; where ZT 0= zeitgeber time 0, time of lights ON). In the following week, the food was given 2 hours after ‘lights on’ (TII; ZT 2–4, 6–8, 10–12). The food intake and locomotor activity under each condition were observed.

**Results:** The results showed that locomotor activity was induced during food deprivation and suppressed during food availability. Also the food deprivation led to increased food intake.

**Conclusion:** Our results suggest that intermittent food availability/deprivation reallocates the locomotor activity and food intake in Spotted Munia.

## Background

Different environmental factors such as photoperiod, temperature, food availability, species interaction/competition and predation influence birds’ decision of timing and duration of different activities to maximize their fitness [1,2,3,4]. These decisions affect their behavior in space (e.g. distribution; [5]) and time (e.g. daily and seasonal activities; [6,7]).

Among all behaviors, the pattern of daily feeding and locomotion is mostly affected by these environmental factors [8,9]. Generally, feeding and locomotion show high activity in the morning and evening, presenting a bimodal pattern [10,11,12,13]. This is because most of the birds adjust their feeding pattern from higher food intake at risk prone foraging time in the morning to low intake at low risk time in the evening [14]. When food supply is abundant, the foraging interruptions during day are scheduled possibly to minimize predation risk; however, in low food availability birds are compelled to forage continuously throughout the day [11].

The activity pattern may switch from bimodal to unimodal and vice versa [15,11] and depends upon season [6]. Mostly, the time allocation of these activities depends on hunger state of the individuals [14] or food availability [16,13]. However, temperature [17,18] and avoidance of predators [19,11] also affect their pattern.

In nature, locomotion and feeding are interrelated behaviors. In unpredictable food conditions, birds may have to explore for longer time and in a larger area, which may affect their day-time rest period. This may increase the risk of foraging and alter the balance between energy maintenance and foraging [20]. In such situations birds may respond differently; they may increase the time allocation for food procurement or lower the exploratory activity to maintain the same energy. Such changes in behavioural responses are likely to affect fitness of the individuals in two ways: the increased time allocation for foraging would affect their social interaction and increase the predation risk, and increased fitness costs would affect the rate of energy gain [21,22,23].

Several studies using food manipulation protocols such as time and duration [24], amount [25] and the interruptions to foraging [26] have demonstrated its effect on circadian and seasonal responses. If an animal is denied access to food, the motivation to get it becomes stronger or may be exaggerated resulting into changed intensity and time allocation for other behaviours [27]. For example, food deprivation in meadow voles affected the sexual behavior and inhibited their perceptivity and receptivity [28], whereas in red jungle fowl and white leghorn layers it induced more foraging-exploring and less preening-perching behaviors [29]. In birds and mammals, the variation in food availability or interruptions in foraging directly affects their ability to regulate energy usage. It affects their body mass [30,31], body fattening [31,32] and behavioral activity and daily torpor [26,25]. Food restriction imposed on quail chicks, either by reducing the amount of food offered or limiting the time, affected the body mass gain [33]. In timed food restriction condition, the birds would increase their food intake either by hoarding externally or internally in their crops [34,35].

Various studies have demonstrated the effect of food on daily activity pattern in temperate and tropical birds (36–42). However, none of these studies ask how different behaviors may interact in a situation of food deprivation. Therefore, the present study aimed to find out the effect of food deprivation on two interrelated behaviors, the feeding (food intake) and locomotion in Spotted Munia (*Lonchura punctulata*).

## Methods

This study was conducted on adult Spotted Munia (*Lonchura punctulata*), a tropical passerine finch from family Estrildidae. Birds were captured from the nearby areas of Lucknow (26°55’N, 80°59’E) in July 2010, and maintained in groups in an outdoor aviary under natural photoperiod. Acclimated birds (*N* = 12) were housed singly in activity recording cages (size = 60 x 45 x 35 cm) placed inside the photoperiodic chambers lit by compact fluorescent bulbs. All photoperiodic chambers were isolated from each other. Each activity cage had two perches, and from outside was mounted with an infrared motion detector (IR sensors; Conrad, CK Intellisense, Germany), and food and water cups. Each IR sensor was connected to a separate channel of computerized data recording system, which collected general activity of bird in the cage in 5 min bins. The collection and analyses of activity data were done using Chronobiology Kit software (Stanford Software Systems, USA).

Birds were exposed to equinox photoperiod (12L:12D; 12h light: 12h dark; *L* = 350 ± 20 lux; *D* < 1 lux) and constant temperature conditions (24 ± 2 °C). They were randomly divided into two groups (*N* = 6 each). They were fed on seeds of *Setaria italica* and *Oryza sativa*. The water was given *ad libitum*. Group 1 birds were given food *ad libitum* (Control; C) for the entire duration of experiment but group 2 birds were given restricted (treatment; T) feeding schedules as follows: food *ad libitum*, day (1–7) followed by two hours food presence (P) alternating with two hours of food absence (A) (2P:2A; TI; day 8–15 and TII; day 16–22). In TI, the food was available with ‘lights on’ (TI; ZT 0–2, 4–6 and 8–10; ZT 0= zeitgeber time 0, time of lights ON) for a week whereas in the following week, in TII it was available 2 hours after ‘lights on’ (TII; ZT 2–4, 6–8, 10–12).

Thus, the two treatments (TI and TII) had food for 6 hours in a 12 h light period, though differed in its timing. In these groups, the food intake was measured as follows: The food filled cups were given at ZT 0–2, 4–6, 8–10 (TI) and ZT 2–4, 6–8 10–12 (TII). During no food condition, the filled food cups were replaced by empty cups. The replacement of food cups was done simultaneously in control group also, to control for the effect of food handling on locomotor activity pattern. Food intake during two hours of its availability (in both TI and TII) was measured as: food given - food left including husk. Simultaneously, the food intake for every two hours from ZT 0–2, 2–4, 4–6, 6–8, 8–10 and 10–12 in control group was also measured to make comparison between corresponding values at ZT 0–2, 4–6 and 8–10 (CI vs TI) and ZT 2–4, 6–8 and 10–12 (CII vs TII). The animal care and procedures adopted in this study were as per guidelines of the Institutional Animal Ethics Committee (IAEC).

## Statistical analyses

The hourly activity counts and total counts in a 24 h day were plotted as mean ± SEM for each group. The difference in the activity pattern within a group across the day was analyzed by one-way analysis of variance with repeated measures (one-way RM ANOVA). The difference in activity counts or food intake among treatments was analyzed by one-way ANOVA. Two way ANOVA using Bonferroni *post* test was used to analyze the difference between two treatments, considering food condition as factor 1 and time as factor 2. The significance in activity counts or food intake per day between two food conditions was tested by Student’s t-test. All the statistical analyses were done using GraphPad Prism software ver. 5.0 (GraphPad Software, San Diego, CA).

## Results

**Figure 1:**
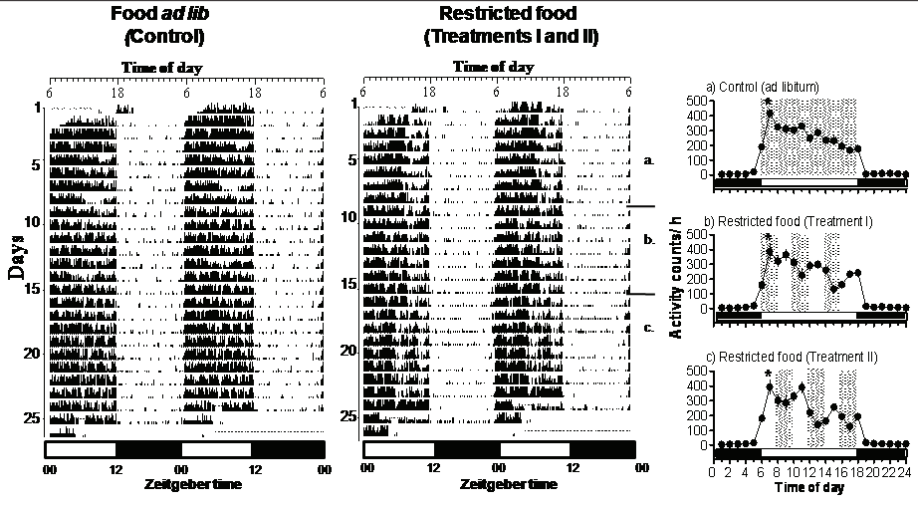
Representative actograms of the Spotted Munia kept under different food conditions (*ad libitum* = control, left panel and restricted feeding (Treatments I and II), middle panel). Right panel shows the activity profile across 24 h day under different food conditions for the number of days marked as a, b and c on the right actogram. Bars below each actogram show the light:dark (12L:12D) cycle. The time of lights ON is marked as ZT 0. In the activity profile the hashed area shows the time of food availability. * indicates the significance at p < 0.0001, one-way RM ANOVA.

**Figure 2:**
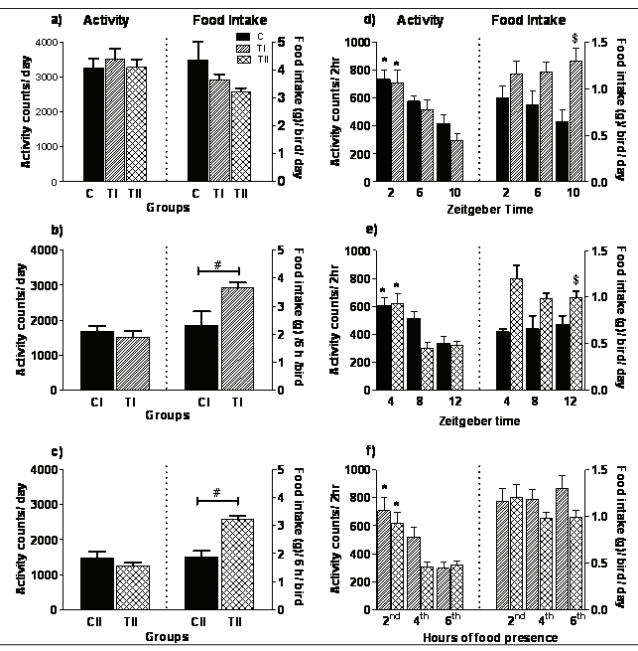
Mean (± SEM) activity counts and food intake in Spotted Munia held under different food conditions. (a) total counts and food intake per day in control (C) and different food conditions (TI and TII). (b) activity counts and food intake in 6 hours of food availability in treatment I (TI) and corresponding hours in *ad libitum* group (CI) (c) and in treatment II (TII) and corresponding hours in *ad libitum* group (CII) (d) activity counts and food intake during ZT 0–2, 4–6 and 8–10 (TI) and corresponding hours in control group (CI) (e) and during ZT 2–4, 6–8 and 10–12 (TII) and corresponding hours in control group (CII), (f) activity counts and food intake in the hours of food presence in both the treatment groups (TI and TII). # indicates significance at p < 0.05; Student’s unpaired *t*-test, * indicates time dependent effects and $ indicates food treatment dependent effects, significance at p < 0.0001; two-way RM ANOVA (Bonferroni *post* test).

Figure 1 shows representative actograms of birds under different food conditions. Birds in all conditions displayed diurnal activity (C: F_5,23_ = 61.22, p < 0.0001, TI: F_5,23_ = 44.44, p < 0.0001 and TII: F_5,23_ = 42.93, p = 0.0003; one-way RM ANOVA, Figures 1a-c). During differing food conditions (TI and TII), the hourly activity profile showed significantly low and high counts across the day (during light hours) but under food *ad libitum* (C) it declined gradually and significantly from dawn to dusk (Figures 1a-c). The mean counts per day, however, in all the three conditions were similar (F_2,14_ = 0.2389, p = 0.7907, one way ANOVA, Figure 2a).

Though, the total counts in the period of food availability every two hours (total 6 h/day) in both TI and TII showed no difference with the corresponding hours in control group, CI and CII respectively (Figures 2b and c), it showed time dependent effect on distribution of locomotor activity (CI vs TI; food conditions: F_1,18_ = 0.7544, p = 0.4076; time: F_2,18_ = 34.20, p < 0.0001 and interaction [food condition x time]: F_2,18_ = 0.6146, p = 0.5518; CII vs TII; food conditions: F_1,18_ = 1.906, p = 0.2007; time: F_2,18_ = 35.72, p < 0.0001 and interaction [food condition x time]: F_2,18_ = 0.1075, p = 0.8987; two way ANOVA Figures 2d, e). Irrespective of the timing of food availability, the locomotor activity in both TI and TII showed time dependent effects (TI vs TII, food conditions: F_1,20_ = 0.9792, p = 0.3457; time: F_2,20_ = 29.79, p < 0.0001 and interaction [food condition x time]: F_2,20_ = 2.042, p = 0.1559; two-way ANOVA, Figure 2f).

The total food intake/day/bird also showed no difference amongst food conditions (F_2,14_ = 3.074, p = 0.0782 one-way ANOVA, Figure 2a). However, in TI and TII it was significantly high compared to the corresponding hours in control group, CI and CII, respectively (CI vs TI: p = 0.0305; CII vs TII: p = 0.0008; Student’s unpaired t-test, Figures 2b and c). It showed food condition, but not time, dependent effect (CI vs TI; food conditions: F_1,18_ = 10.15, p = 0.0111; time: F_2,18_ = 0.09961, p = 0.9057 and interaction [food condition x time]: F_2,18_ = 1.679, p = 0.2144 and CII vs TII; food conditions: F_1,18_ = 27.85, p = 0.0005; time: F_2,18_ = 0.8580, p = 0.4406 and interaction [food condition x time]: F_2,18_ = 1.720, p = 0.2072; two-way ANOVA, Figure 2d and e). The food intake in both TI and TII did not show any food condition or time dependent effect (food conditions: F_1,20_ = 3.204, p = 0.1037; time: F_2,20_ = 0.4661, p = 0.6341 and interaction [food condition x time]: F_2,20_ = 1.439, p = 0.2606; two-way ANOVA, Figure 2f).

## Discussion

Prolonged food deprivation may lead to loss of energy reserves [43]. This motivates energy allocation towards the physiological functions to sustain life [43]. As a result, in a condition of food deprivation followed by abundance, most organisms show ‘compensatory hyperphagia’ to replenish the energy reserves [44]. Our results also showed that food interruption affected the allocation of locomotion and feeding behaviors in Spotted Munia. During intermittent food deprivation, the activity levels increased indicating the motivation to explore. This could be due to the fact that hungry animals display more effort to get food and this motivation is associated with the biological relevance the animal has assigned to the goal (e.g. food in this case) [45]. However, when food was available, this motivation shifted from exploration to feeding and resulted in decreased activity levels.

Our results showed that the total food intake was similar in both control (food available for 12 h) and treatment (food available for 6h) feeding schedules (Figure 2a). This suggests that food availability followed by food deprivation induced higher intake. The food intake in birds, in response to interrupted feeding schedules, depends on their energy needs as determined by the length of fasting prior to feeding [33].

The time allocation decisions that are dependent on hunger state of the individual may change its daily routine. As a result, birds having negative energy budget in the morning would shift their foraging behavior from risk-sensitive to risk-averse in the evening, when they have positive energy budgets [46,47]. The time allocation decisions were also related to feeding patch and flock size. Cranes showed higher locomotor activity during the morning when food availability was higher and easily obtained than in the evening when food was not easily available [14]. It has been shown that animals may regulate their energy budgets either by altering energy expenditure on a particular activity or by selecting an alternative behavior that differs in its energy costs [48].

In our study, no difference in the total activity counts and food eaten per day in all the feeding schedules indicated that the two behaviors were temporally allocated. Such reallocation seems to be an innate tendency that helps the animal to alter its behaviors in response to environmental challenges [49]. Our results are in agreement with other studies on birds shown to motivate foraging and exploration in unpredictable and variable food conditions [50]. In quail, restricted feeding resulted in higher levels of locomotor activity than controls [33]. The oystercatchers (*Haematopus ostralegus*) given food for a shorter time increased their food intake to maintain the same mean consumption over a longer period [51]. In chickens, the food deprivation by half or three-fourth of the normal amount than they would take in *ad libitum* condition increased their feeding motivation. They became differentially sensitive to different levels of food availability and showed a linear increase in food consumption with the duration of food deprivation [52,53]. The chickens that experienced more food restriction reacted faster to the food available [54] and vocalized more [55].

In mammals also, food deprivation increases motivation to explore [56,57]. The food deprived sheep showed increased feeding motivation leading to increased exploration [58]. The feeding motivation also changed the ranging pattern in lion tailed macaques [59]. Rats and hamsters showed prolonged and pronounced overeating after food deprivation; however, after an initial increase in food intake there was increase in the food hoarding [60]. In golden hamsters the acute food deprivation increased the feeding latency and speed of eating but did not increase the total food intake [61].

Food restriction can stimulate anticipatory activity, the amount of locomotor activity [30,42], plasma metabolites [62] and cognitive functions [63]. It induces changes in the metabolic hormones (leptin and ghrelin). In mammals including humans, it decreases circulating leptin but increases ghrelin levels [64]. Ghrelin stimulates the appetite via the hypothalamus [65]. The effect of food deprivation seems to influence the neuronal circuitry also. In hamsters, different patterns of c-fos reactivity in the amygdala have been observed during food absence/presence conditions [66]. The c-fos cell counts increased with time when there was no food but the locomotor activity decreased. This decrease in activity could be due to the energy-conservation strategy at a time when no food is easily available.

In summary, increased exploration and food intake without hoarding during the periods of food deprivation and availability, respectively, suggests that birds may have an energy dynamics different from what has been reported in mammals. Our results also suggest that the two behaviors (feeding and locomotion) are reallocated temporally by the environmental constraint (food deprivation). Further studies could investigate whether this trade-off changes seasonally.
